# Toward Standardized Monitoring of Patients With Chronic Diseases in Primary Care Using Electronic Medical Records: Development of a Tool by Adapted Delphi Procedure

**DOI:** 10.2196/14483

**Published:** 2020-03-25

**Authors:** Leandra Falck, Marco Zoller, Thomas Rosemann, Nahara Anani Martínez-González, Corinne Chmiel

**Affiliations:** 1 Institute of Primary Care University of Zurich and University Hospital of Zurich Zurich Switzerland

**Keywords:** monitoring of chronic diseases, indicators, primary care, electronic medical record, diabetes mellitus type 2, arterial hypertension, asthma, osteoarthritis, chronic heart failure

## Abstract

**Background:**

Long-term care for patients with chronic diseases poses a huge challenge in primary care. There are deficits in care, especially regarding monitoring and creating structured follow-ups. Appropriate electronic medical records (EMR) could support this, but so far, no generic evidence-based template exists.

**Objective:**

The aim of this study is to develop an evidence-based standardized, generic template that improves the monitoring of patients with chronic conditions in primary care by means of an EMR.

**Methods:**

We used an adapted Delphi procedure to evaluate a structured set of evidence-based monitoring indicators for 5 highly prevalent chronic diseases (ie, diabetes mellitus type 2, asthma, arterial hypertension, chronic heart failure, and osteoarthritis). We assessed the indicators’ utility in practice and summarized them into a user-friendly layout.

**Results:**

This multistep procedure resulted in a monitoring tool consisting of condensed sets of indicators, which were divided into sublayers to maximize ergonomics. A cockpit serves as an overview of fixed goals and a set of procedures to facilitate disease management. An additional tab contains information on nondisease-specific indicators such as allergies and vital signs.

**Conclusions:**

Our generic template systematically integrates the existing scientific evidence for the standardized long-term monitoring of chronic conditions. It contains a user-friendly and clinically sensible layout. This template can improve the care for patients with chronic diseases when using EMRs in primary care.

## Introduction

Long-term care for patients with chronic diseases poses a huge challenge. There are deficits regarding monitoring and creating structured follow-ups. In Switzerland, unlike other countries, there are a plethora of different electronic medical record (EMR) providers. Although no official registry is maintained, the estimated number of current EMR providers is 60. Due to the lack of unified software standards, interaction between providers is impossible and migration of data is practically unfeasible. This fact illustrates the need for standardization across EMRs, and this may also be the reason why many practices in Switzerland still prefer paper-based records.

To introduce a tool for monitoring patients with chronic diseases within the EMR, it is essential to know the needs of potential users and to develop a customized tool. A survey of physicians not using EMRs showed that most concerns relate to the improvement of quality of care, the workflow process, and the physician-patient relationship [1-3]. Thus, to increase EMR use, it is crucial to enhance the benefits. This can be achieved by customizing the EMR for increased productivity. For example, combining monitoring elements with time saving features, ergonomic navigation, and clear design could facilitate fast retrieval of all relevant information.

EMRs are not only a practical bookkeeping tool; they can also improve disease management. For chronic diseases, EMRs enable more thorough record keeping and surveillance of treatment intensification, thus improving monitoring [4-6]. In addition, EMRs can help with both documenting and reducing errors that are common in paper-based medical records, such as legibility, prescription, and transcription errors [7,8]. Further advantages of EMRs include the graphic representation of monitoring indicators and, more importantly, the migration of data for care coordination between different providers and between providers and patients. However, the poor dissemination and lack of standardization of EMRs poses a huge obstacle for research in primary care.

We have previously identified a structured set of evidence-based indicators for five common chronic conditions [9]. In this study we aimed to develop an evidence-based standardized, generic template that improves monitoring of patients with chronic conditions in primary care by means of an EMR.

## Methods

**Figure 1:**
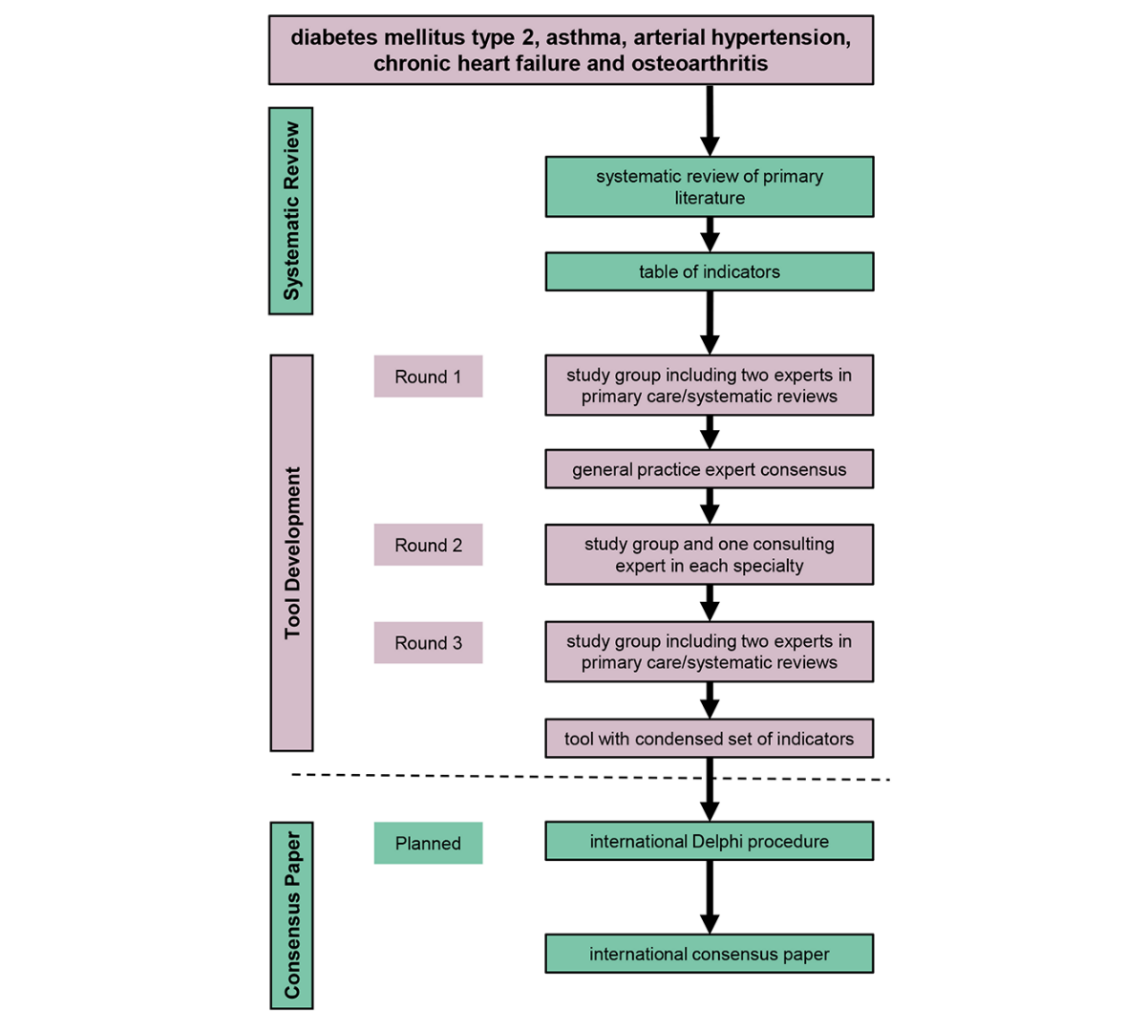
Study flow: Summary of all steps of the monitoring tool project.

Figure 1 shows an overview of the methodology.

### Prior Work: The Systematic Review

In the first phase of this study [9], we performed a systematic review to identify and assess a set of clinical indicators that can be used for monitoring chronic diseases in primary care. In this study, we selected clinical indicators for five diseases that have high prevalence globally and are associated with multimorbidity and polypharmacy including type 2 diabetes mellitus, arterial hypertension, chronic heart failure, asthma, and osteoarthritis [10,11]. The review consisted of the appraisal of clinical guidelines and primary peer-reviewed studies of any design that were carried out mainly in primary care.

### Tool Development

The second phase in this study was an adapted Delphi procedure conducted to evaluate the usability of the indicators in practice and to develop a monitoring tool by integrating the indicators in a user-friendly EMR layout. Figure 1 presents the three phases of the study and the associated research steps. The steps for the development of the tool included consensus from general practice experts, an evaluation by specialists, and a draft of a tool that included the relevant indicators. In the following sections, we present the methods in more detail.

### General Practice and Expert Consensus

Experts from the region of Zurich were contacted by email. We identified “well-renowned” expert physicians in the region of Zurich that had a special interest in chronic care and work experience in an outpatient setting. The experts were either well-known by some members of our team, who are clinically active physicians, or were identified by asking colleagues. All contacted experts agreed to participate. The study team included two experts in primary care and EMRs (CC and MZ) and an expert in systematic reviews (NM-G) as well as a medical student who was not considered as an expert (LF). For asthma and arterial hypertension, two experts in outpatient care with a special interest in chronic care, Dr. Claudia Steurer-Stey and Dr. Paolo Suter, participated in the study. For type 2 diabetes mellitus, chronic heart failure, and osteoarthritis, Dr. Henryk Zulewski, Dr. Tobias Höfflinghaus, and Dr. Lukas Wildi, PD participated, respectively. We contacted five additional experts in total, one for each chronic disease. Multimedia Appendix 1 shows a list of all experts and affiliations.

### Assessment of the Monitoring Indicators

Experts in any of the five chronic diseases evaluated the set of indicators by means of an adapted Delphi procedure. The Delphi procedure is a structured communication method consisting of a panel of participants and experts on a certain topic [12]. Multiple rounds are conducted on a specific topic, and in every new round the decision of one expert is influenced by the anonymous decisions of the rest of the expert panel in the previous round. The Delphi procedure has been proven to be a feasible method in evaluating indicators for chronic diseases and for generating a consensus [13,14]. We use the term *adapted Delphi procedure*, because the evaluation rounds were not anonymous and consisted of only a few participants. In total, we performed three rounds.

The first round consisted of face-to-face meeting sessions with our study group of four, including two experts in primary care and EMRs, one expert in systematic reviews, and a doctoral student (Multimedia Appendix 1). Each indicator was categorized into one of four types of data elements: 1) to be part of the monitoring at least annually, 2) data normally included in EMRs, 3) data not to be collected at a regular basis, and 4) data that should not be collected at all. Table 1 exemplifies this approach for diabetes mellitus. Before the second discussion round, we excluded data the experts indicated should not be collected.

**Table 1:** Extract from the set of indicators for diabetes mellitus type 2 identified by the systematic review and categorized based on the adapted Delphi procedure.

Indicators	Considered to be part of the monitoring at least annually	Data normally included in EMR	Data not to be collected at a regular basis	Data that should not be collected
Diabetes education history	X	—^a^	—	—
Current treatment	—	X	—	—
Weight history	—	—	—	X
Vaccination status	—	X	—	—
Physical activity patterns	—	—	X	—
Heart rate	—	—	—	X
ECG	—	—	—	X
Self-monitoring of urine glucose	—	—	—	X
Nutritional status	—	—	—	X
Teeth’s condition	—	—	X	—
Eye examination	X	—	—	—
HbA_1c_	X	—	—	—
Inspection of skin	X	—	—	—
Hyperkeratosis	X	—	—	—
Dryness	X	—	—	—
Dilated veins	X	—	—	—
Skin examination for insulin injection sites	X	—	—	—

^a^Not applicable.

In a second round, the study group and one consulting expert of each specialty discussed the condensed set of indicators resulting from the first round (Multimedia Appendix 1). The discussion led to an even more condensed set of indicators, which the study group further re-evaluated in a third round to focus on feasibility and exclusion of redundant indicators.

### Design and Development of the Monitoring Tool

Based on a table of the condensed set of indicators, we developed a framework table for each condition, including 1 to 4 sublayers, to provide a structure for the indicators. We introduced the different layers to optimize usability and improve the overview. Layer one is the first visible layer when the tool opens. Each indicator represents itself or its own category of sub-indicators. When an indicator is selected from the first layer, the subsequent layer becomes visible. We designed the monitoring tool in a layout format that enables its integration into an EMR.

## Results

### Delphi Procedure

The adapted Delphi procedure resulted in a thorough set of indicators, since only relevant and practical (ie, useful and operable) indicators were selected. The systematic review provided 1162 indicators for the five chronic conditions; however, only 25.47% (296/1162) were considered by the experts as being of high enough relevance and feasibility to be implemented in the monitoring tool, including 20.48% (51/249) of diabetes mellitus indicators (ie, 12 in additional tab, 4 in cockpit, 11 in first layer, 21 in second layer, and 3 in third layer), 26.78% (49/183) of asthma indicators (ie, 12 in additional tab, 3 in cockpit, 7 in first layer, 11 in second layer, and 16 in third layer), 14.63% (49/335) of arterial hypertension indicators (ie, 12 in additional tab, 4 in cockpit, 3 in first layer, 23 in second layer, and 7 in third layer), 33.33% (77/231) of chronic heart failure indicators (ie, 12 in additional tab, 6 in cockpit, 6 in first layer, 40 in second layer, and 13 in third layer), and 42.68% (70/164) of osteoarthritis indicators (ie, 12 in additional tab, 7 in cockpit, 7 in first layer, 17 in second layer, 20 in third layer, and 7 in fourth layer).

### The Monitoring Tool

**Figure 2:**
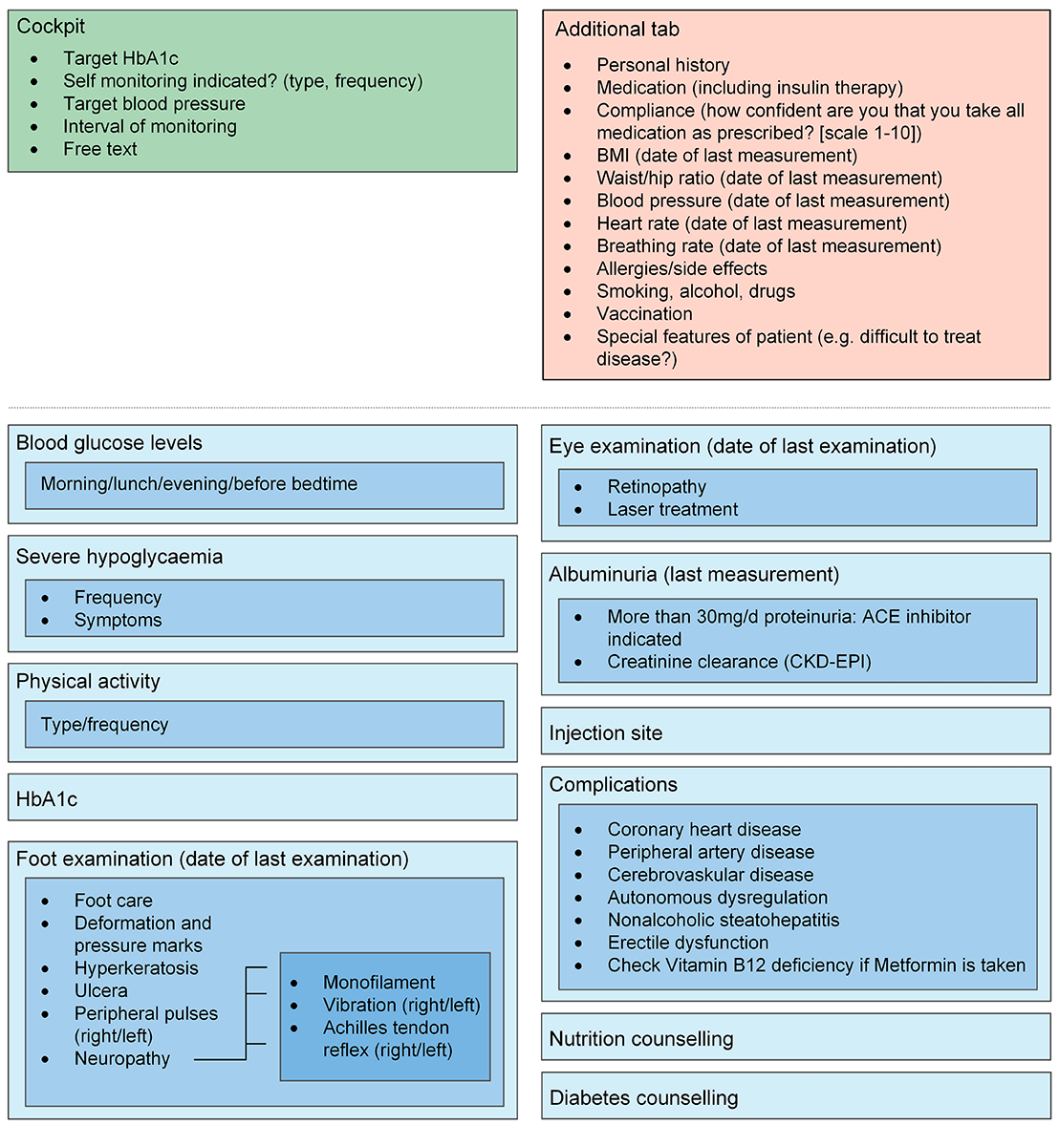
Monitoring of diabetes mellitus type 2. Light blue to dark blue represents layers one to three. HbA_1c_: glycated haemoglobin; ACE: angiotensin-converting enzyme; CKD-EPI: Chronic Kidney Disease Epidemiology Collaboration.

**Figure 3:**
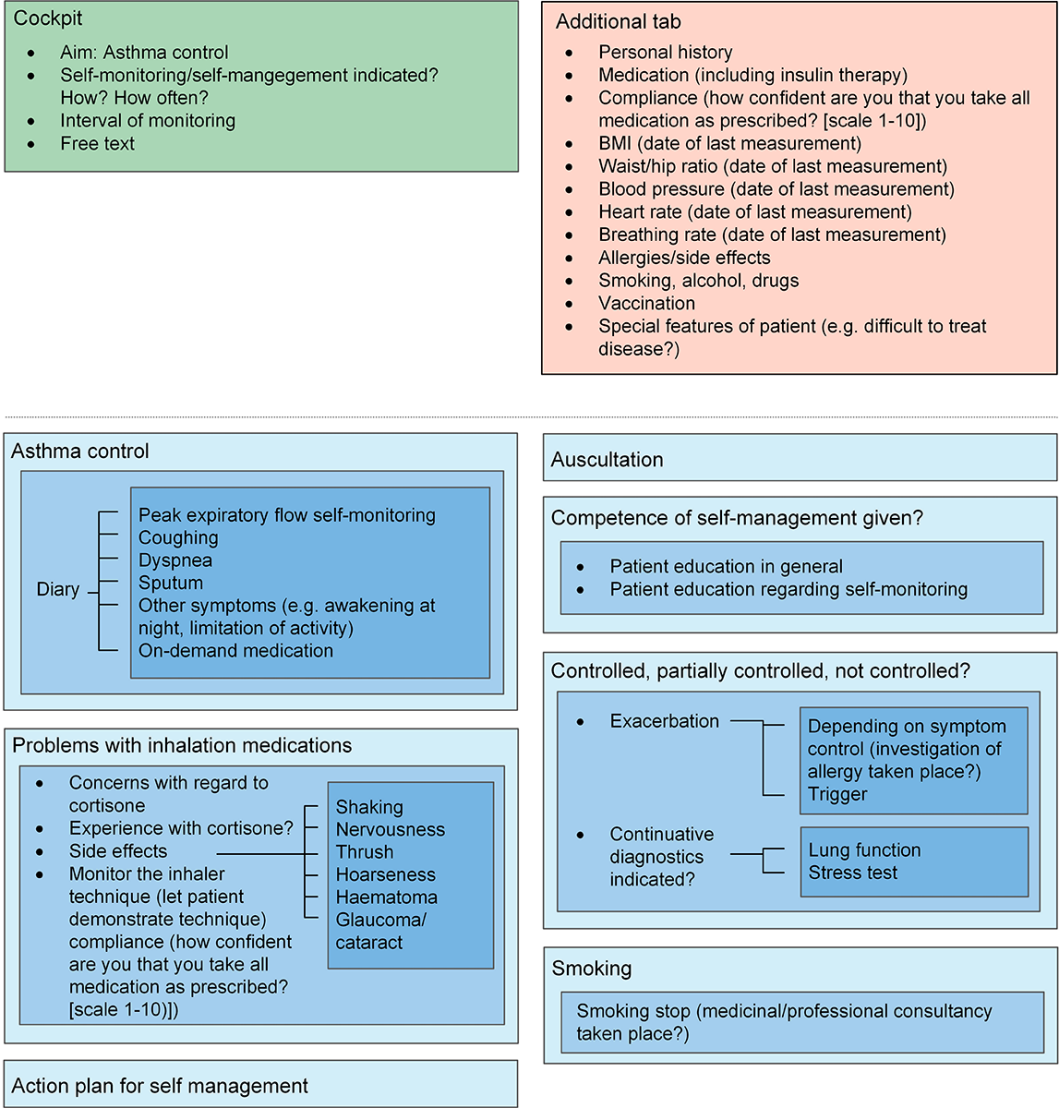
Monitoring of asthma. Light blue to dark blue represents layers one to three.

**Figure 4:**
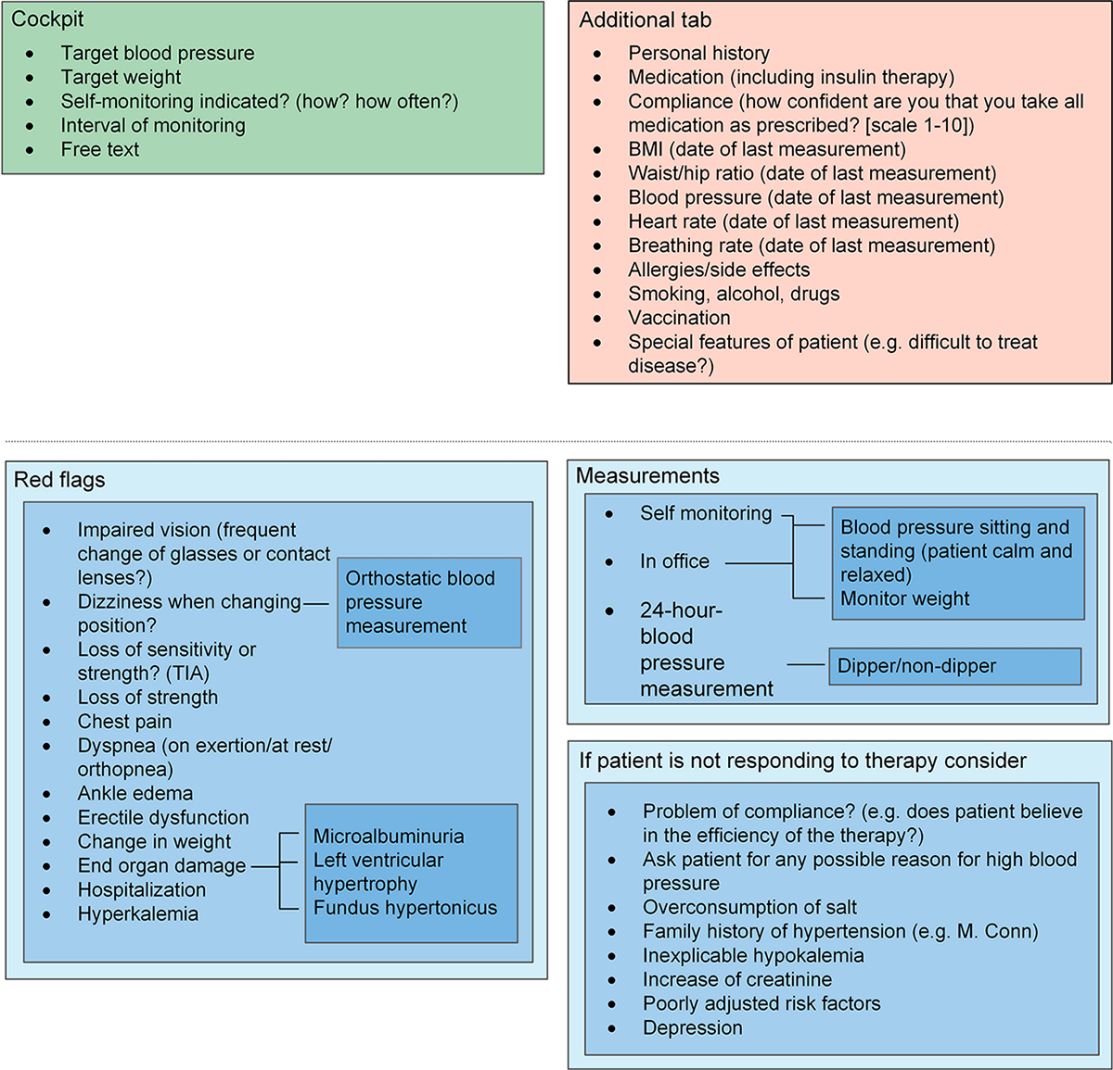
Monitoring of arterial hypertension. Light blue to dark blue represents layers one to three. TIA: transient ischemic attack.

**Figure 5:**
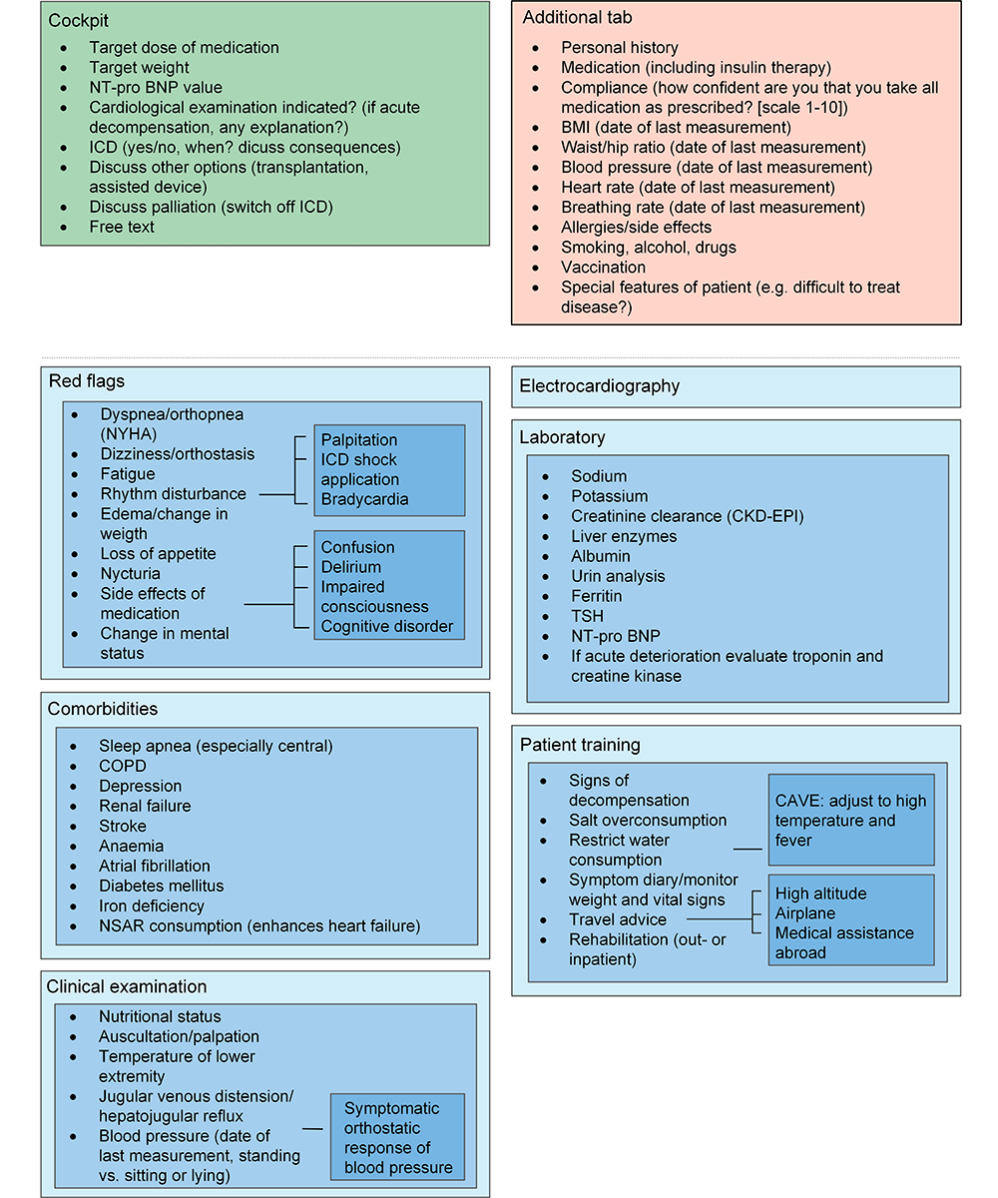
Monitoring of chronic heart failure. Light blue to dark blue represents layers one to three. NTproBNP: N-terminal pro b-type natriuretic peptide; ICD: International Classification of Diseases; NYHA: New York Heart Association; COPD: chronic obstructive pulmonary disease; NSAR: nonsteroidal antirheumatics; CKD-EPI: Chronic Kidney Disease Epidemiology Collaboration; TSH: thyroid stimulating hormone.

**Figure 6:**
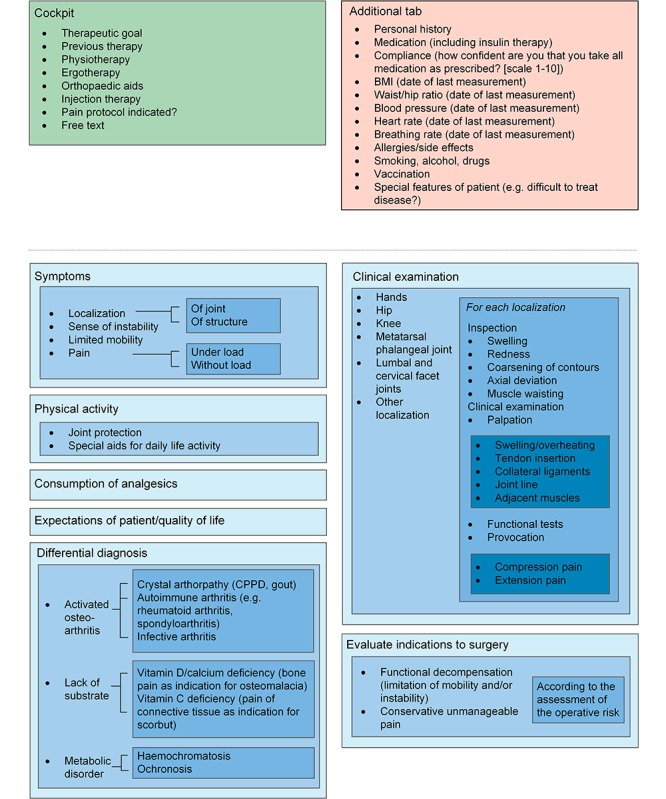
Monitoring of osteoarthritis. Light blue to dark blue represents layers one to four. CPPD: calcium pyrophosphate dihydrate.

**Figure 7:**
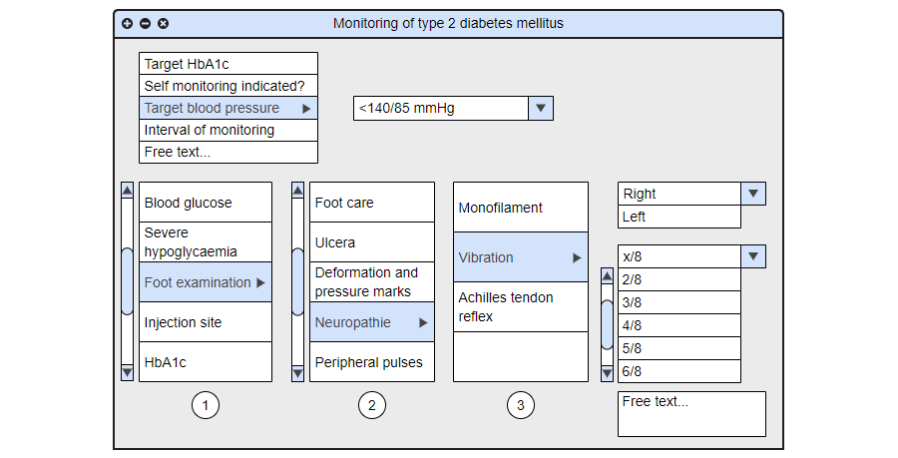
An example of how indicators (here type 2 diabetes mellitus) could be integrated into EMR. Numbers 1-3 represent layers one to three. The top left box is the cockpit. The image has been generated by Moqups (S.C Evercoder Software S.R.L.).

The face-to-face discussion sessions about the eligibility and relevance of the indicators resulted in a condensed set of relevant and practical indicators as part of an EMR monitoring tool. During the Delphi procedure, which was primarily meant to discuss the relevance and feasibility of the indicators, an additional subject that was determined to be important was the ergonomics of how the indicators should be displayed. Ergonomics within the tool were uniformly identified by all involved specialists as an essential element to achieve acceptance of a new monitoring tool. For each selected disease, the indicators were categorized and linked into sublayers, which can be accessed depending on the requirements of the user. The first layer gives an overview of the most important indicators or categories. Clicking on each layer opens a set of further indicators. For a clear design and ergonomic use, we did not exceed four layers. The tool contains an additional tab with nondisease-specific information such as allergies, smoking, or drinking habits. This tab can be accessed at any point during tool use. The tool is completed by a “cockpit”, which serves as a guidance in the process of disease management. The cockpit includes individually predefined treatment goals, and thus enables benchmarking in the monitoring process. In addition, the monitoring interval can also be documented. To guarantee individual adjustments, blank spaces described as “free text” are added in every layer. Figures 2-6 show the final design of the suggested monitoring tool that evolved during the Delphi procedure. Figure 7 shows an example of how some of the indicators of type 2 diabetes mellitus could be integrated into the EMR.

## Discussion

To our knowledge, this study represents the first scientifically-based recommendation for the generic, standardized long-term monitoring of patients with chronic illnesses in primary care by means of a user-friendly and clinically sensible EMR layout. Previously, Steenkiste et al [13] used an adapted Delphi procedure to identify items for diagnosis and treatment of asthma for a Dutch electronic patient record. The adapted Delphi procedure proved to be a feasible method for selecting the indicators. However, a complete list of the items or a graphical presentation of the items is lacking in their study. Similar to our study, Lougheed et al [15] used the Delphi procedure and modified RAND/UCLA Appropriateness Method to identify core and optional data elements for asthma and chronic obstructive pulmonary disease to be integrated into electronic health records (EHR) for primary and tertiary care. In contrast to our study, their selection of indicators was not based on a systematic review but on several data sources. Their method also lacked a presentation of potential implementation.

The tool that we developed facilitates the monitoring of patients with chronic diseases by providing all the essential monitoring indicators, which should be assessed at regular intervals according to the current scientific evidence. All indicators are arranged in up to four sublayers that contain only the most relevant indicators. This layout avoids a surplus of information and ensures that the patient, not the computer screen, remains the focus of the consultation. Therefore, all sublayers exceeding the first one are only visible if actively clicked on. The cockpit gives an overview of all preset goals as well as the current situation and, therefore, can serve as a benchmarking tool. By clearly displaying this critical information, the cockpit facilitates a patient handover or holiday replacement and, therefore, enables the continuity of care. Preset goals also help to overcome clinical inertia, a widespread problem in the care of patients with chronic diseases [16]. Additional tabs that contain nondisease-specific information, such as allergies or body mass index, give an overview on basic but relevant patient information.

In Switzerland, where the study was conducted, EMRs are less developed than in other countries, and the number of physicians still using paper-based medical records is higher than elsewhere [1,3]. Therefore, it is necessary to first provide a functioning EMR basis within primary care. In a second step, it will be desirable to integrate modern applications into the EMR, such as mobile devices that allow patients to be in more control of their chronic conditions. The standardization of EHR tools such as the ones presented here could enable the provision of decision-support tools and add an extension to an EHR. This will link physicians and patients to provide a holistic approach to the process of monitoring.

In long-term care, the involvement of several professionals of different health care disciplines is common. Skill-mix models involving nonphysician disciplines, such as practice nurses, dieticians, or physiotherapists are on the rise; however, due to the specific regulations in different countries concerning allocation of responsibilities, it is not feasible to establish an international standard. This tool will thus have to be adapted according to different health care systems and their needs.

### Strengths and Limitations

A strength of this project is the iterative Delphi process that identified the importance of the ergonomic layout of the monitoring tool. Consideration of ergonomics can enhance user-friendliness and facilitate chronic care within an EMR. This tool offers a practical approach for implementing scientific results into everyday practice. By involving generalists with extensive practice experience as well as specialists in different medical fields through an adapted Delphi procedure, a condensed set of indicators were identified as relevant for everyday use in primary care. A potential limitation of our study is that the adapted Delphi procedure did not meet all criteria of a typical Delphi procedure. Since the discussions were not performed anonymously and the persons involved might have selected the indicators they were most familiar with, this may have increased the chances of bias. Furthermore, the adapted Delphi procedure included in total only ten experts and, in certain phases, only four experts (three experts from the study group including two in primary care and EMRs and one in systematic reviews, and an additional expert in the respective field), which might reduce external validity. Additionally, the complexity of the technical integration of indicators (beyond the visual layout on the screen) is not addressed in this manuscript. Details of EMR integration will vary between the different software companies and the needs of different health care systems.

### International Consensus Paper

As mentioned in Figure 1, our final goal is an international consensus paper. This will be achieved by an international Delphi procedure with a larger number of experts in general practice and other specialties. To meet the demands of monitoring the rising number of multimorbid patients, the method we present here is meant to be extended to more diseases that will also be linked to each other. In the future, we hope the tool will become an integral part of the process of collecting patient data, as well as a clinical decision support system that will directly link current guidelines and algorithms with therapy suggestions.

### Conclusion

Our generic template systematically integrates the existing scientific evidence for the standardized long-term monitoring of chronic conditions. It contains a user-friendly and clinically sensible layout and can improve the care of patients with chronic diseases by means of an EMR in primary care.

## Appendix

Multimedia Appendix 1 A list of all experts and their affiliations.

## Abbreviations

EHR: electronic health record
EMR: electronic medical record.
