# Supplementary material for: Toward Standardized Monitoring of Patients With Chronic Diseases in Primary Care Using Electronic Medical Records: Development of a Tool by Adapted Delphi Procedure
Source: JMIR Med Inform. 2020 Mar 25;8(3):e14483. doi: 10.2196/14483 (PMC7142740; doi:10.2196/14483)
Supplement: Multimedia Appendix 1 [file medinform_v8i3e14483_app1.docx]

List of involved experts.

Prof. Dr. med Henryk Zulewski for type 2 diabetes mellitus

- Head Department of Endocrinology and Diabetology, Triemli Hospital Zurich
- Faculty of Medicine, University of Basel

Prof. Dr. med. Claudia Steurer-Stey for asthma

- Specialist in internal medicine and pulmonary diseases
- Practicing as a physician in mediX group practice in Zurich and as a researcher at the Epidemiology, Biostatistics and Prevention Institute
- Special interests: chronic care and self-management
- Member of various advisory boards with a focus on chronic care and disease management

Prof. Dr. med. Paolo Suter for arterial hypertension

- Specialist in internal medicine
- Senior physician at the Department of Internal Medicine and Medical Policlinic at the University hospital of Zurich
- Special interests: nutrition and prevention of chronic diseases

Dr. med. Tobias Höfflinghaus for chronic heart failure

- Specialist in internal medicine and cardiology
- Special interests: chronic heart failure

PD Dr. med. Lukas Wildi for osteoarthritis

- Head Department of Rheumatology, Kantonsspital Winterthur, Zurich Senior physician at the Department of Rheumatology at the University Hospital of Zurich when the Delphi procedure was conducted).
- Special interests: Osteoarthritis

MSc, BScN Nahara A. Martínez-González for health services research, electronic medical records and evidence-based methodology

- Research associate at the Institute of Primary Care, University and University Hospital Zurich
- Special interests: health services research, evidence-based methodology (systematic reviews, meta-analyses), chronic care, skills mix, electronic medical records, antibiotic prescribing

PD Dr. med. Corinne Chmiel for primary care, electronic medical records and systematic reviews

- Practicing as a head physician in mediX group practice in Zurich
- Senior research associate at the Institute of Primary Care, University and University Hospital Zurich
- Special interests: Chronic Care, Skill Mix, electronic medical records, health services research

Dr. med. Marco Zoller for primary care and electronic medical records

- Practicing as a physician in a group practice in Zurich
- Senior research associate at the Institute of Primary Care, University and University Hospital Zurich
